# Assessment of Pineal Gland Volume and Calcification in Healthy Subjects: Is it Related to Aging?

**DOI:** 10.5334/jbr-btr.892

**Published:** 2016-02-01

**Authors:** Mehtap Beker-Acay, Ozan Turamanlar, Erdal Horata, Ebru Unlu, Nurdan Fidan, Serdar Oruc

**Affiliations:** 1 Afyon Kocatepe University Faculty of Medicine, Department of Radiology, Afyonkarahisar, Turkey; 2 Afyon Kocatepe University Faculty of Medicine, Department of Anatomy, Afyonkarahisar, Turkey; 3 Hitit University Training and Research Hospital, Department of Radiology, Corum, Turkey; 4 Afyon Kocatepe University Faculty of Medicine, Department of Neurology, Afyonkarahisar, Turkey

**Keywords:** Computerized tomography, pineal gland, calcification, aging

## Abstract

**Purpose:**

The human pineal gland is a small neuroendocrine organ which produces melatonin. The main goal of this study was to provide a reference range for pineal volume in all age groups and to determine calcified and noncalcified tissue and their proportions, which may be a reflection of melatonin production in all age groups, by using very thin computerized tomography (CT) slices.

**Materials and methods:**

A total of 167 outpatients had undergone cranial CT. Each of the subject’s total pineal volume (TPV), calcified pineal volume (CPV) and noncalcified pineal volume (NPV) according to age groups were calculated in cubic millimeters. Also, proportion of calcification (POC) was noted.

**Results:**

The median values were 88.5 mm^3^ (12.3 mm^3^–411mm^3^) for TPV, 74.3 mm^3^ (12.3 mm^3^–298 mm^3^) for NPV, and 3.9 mm^3^ (0 mm^3^–141 mm^3^) for CPV. POC showed a gradual increase from 0–49 years. In the ≥70 group, when compared with the 60–69 age group, CPV and POC values were significantly lower (P: 0.036, P: 0.034, respectively).

**Conclusion:**

This study brings a radiological point of view to the distribution of pineal calcification according to age that has a link with melatonin secretion.

## Introduction

The human pineal gland is a small crescent-shaped neuroendocrine organ that connects with the diencephalon by the pineal stalk. The pineal stalk has an inferior lip that links the pineal gland to the posterior commissure, and a superior lip to the habenular commissure. The base of the pineal stalk possesses a recess that is related with the third ventricle. It functions in the secretion of melatonin. About 80% of the pineal gland consists of pinealocytes with the main secretory product being melatonin, which has a major role in circadian rhythm [1,2]. Pineal gland calcification (PGC) was demonstrated first by Schüller in 1918 in skull radiographs of autopsy specimens and can be identified elaborately by non-contrast computed tomography (CT) of the brain [3]. The literature from different parts of the world reveals the existence of PGC that increases in prevalence with aging on CT [3,4,5,6]. Calcified glands are correlated with low levels of the urine 6-sulfatoxymelatonin (metabolite of melatonin), denoting a decreased production of melatonin. It was hypothesized that aging is related to pineal failure with the decrease of melatonin [7,8,9,10]. This is the first volumetric CT study providing a reference range for pineal volume in all age groups and determining calcified and noncalcified tissue and their proportions, which may be a reflection of melatonin production in all age groups.

## Methods

### Subjects

A total of 167 outpatients (80 women, 87 men) were recruited for this cross-sectional designated study at our university hospital and it was approved by the local ethical committee.

All patients were selected from outpatient clinics of neurology department with the complaints of predominantly severe headache, syncope or dizziness. Each of the patients had one set of non-contrast cranial CT scans. Depending on the retrospective design of the study we excluded some disorders according to the patients’ hospital records. Exclusion criteria for the patients were: having intracranial mass, cerebrovascular disease, cancer, psychiatric diseases such as Alzheimer’s disease, Parkinson’s disease, bipolar disease or schizophrenia, which can be linked with pineal gland pathology according to the literature [7, 11,12,13,14,15,16,17,18,19,20].

### Multidetector CT Technique and CT Scan Image Analysis

CT examinations were performed on an 80-row detector CT (160 slice) scanner (Aquilion Prime, Toshiba Medical Systems, Nasu, Japan) with the following parameters: collimation, 80 × 2 mm; pitch factor, 0.625; rotation time, 0.75 sec; 120 kVp; 250 mAs, FOV: 220.3 mm and with a slice thickness of 0.5 mm. Adaptive Iterative Dose Reduction 3D (AIDR 3D) was used as an iterative dose reduction software and Sure Exposure 3D was used as a mA modulation software for all the examinations.

**Figure 1 a–c:**
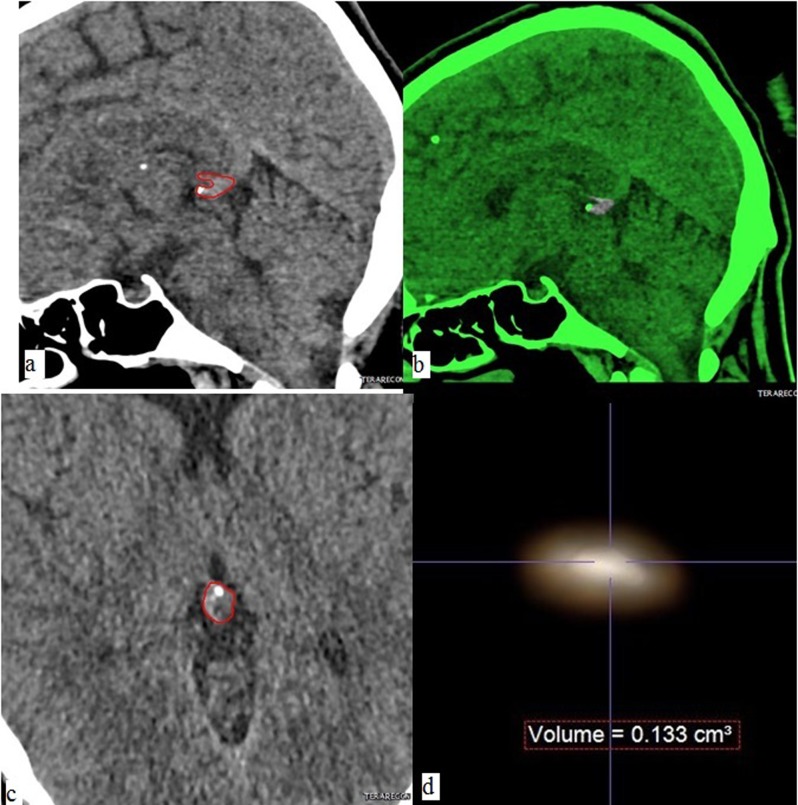
Pineal gland of a 35-year-old female. Image **a** and **b** reveal the outlined pineal gland on sagittal (a) and axial (b) planes on noncontrast computerized tomography images. Green areas on image **c** exhibit the restricted parenchyma by excluding all the calcified tissues from the slices. Image d demonstrates the 3-dimensional image and volume of noncalcifed pineal tissue.

**Figure 2 a–d:**
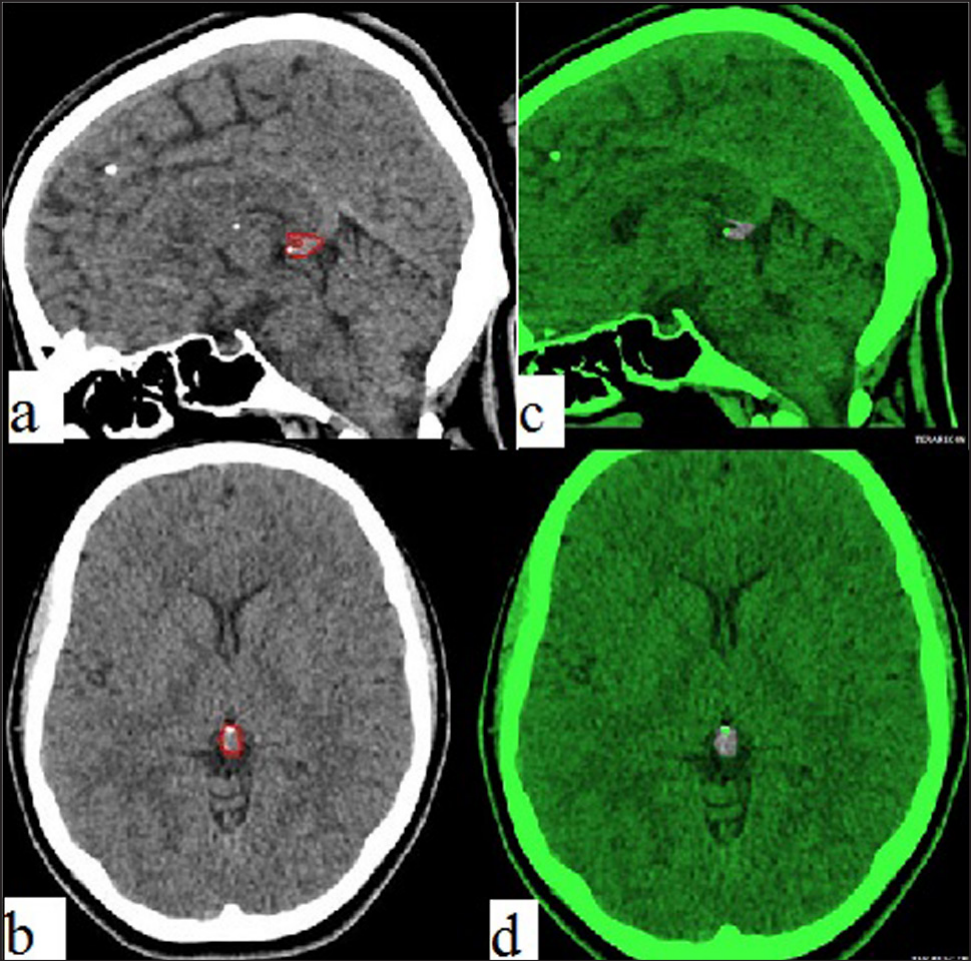
Pineal gland of a 72-year-old male. Image **a** reveals the outlined pineal gland on sagittal plane and image **b** demonstrates the 3-dimensional image and volume of the tissue. Green areas on image **c** and **d** exhibit the restricted parenchyma by excluding all the calcified tissues from the slices.

Axial CT images were transferred to a workstation (Aquarius, TeraRecon Inc., San Mateo, CA, USA). Axial CT, coronal and sagittal MPR images with a slice thickness of 0.5 mm and with section intervals of 0.3 mm were reconstructed with the AquariusNET program (TeraRecon Inc., San Mateo, CA, USA), dividing the computer screen into four equal quadrants in multiview mode. The quadrigeminal cisterna, the mamillary bodies, the ambient cisterna, and the posterior part of the third ventricle were taken into account for anatomical borders for the selection of the pineal region. The pineal gland was outlined using the free hand selection tool, by verifying on axial, sagittal and coronal planes. The appropriate plane was used to calculate the volume in which we could draw the borders of the tissue perfectly and in order to restrict the parenchyma by excluding the pineal recess mostly using the sagittal plane. Then the volume of the outlined region was calculated by computer software, given in cubic millimeters. This analysis was performed on all slices on which the pineal gland, or parts of the pineal gland, were displayed, with 10–35 slices, depending on the measured volume. The noncalcified volume was calculated by excluding the regions more than 70 Hounsfield units. The calcified volume was then manifested by subtracting the noncalcified volume from the total pineal gland volume (Figures 1–2). Proportion of calcification (POC) was given as the proportion of the calcified tissue volume to the total volume in percentage. In order to reduce blooming artifacts that severe calcification may cause, we used very thin slices with optimal collimation. All evaluations were performed blinded by a radiologist (MBA) with at least five years of independent experience.

### Statistical Analysis

For statistical analysis, SPSS statistical software (Version 20.0. Armonk, NY: IBM Corp) was used. Continuous variables are presented as mean and standard deviation, and categorical variables are expressed as percentage. Kolmogorov–Smirnov test was used to evaluate the distribution of variables. Student *t* test was used for continuous variables with normal distribution, and Mann–Whitney U test was used for continuous variables without normal distribution. Volumes among different age groups were analysed using the Kruskal–Wallis test. The correlation coefficients and their significance were calculated using the Pearson test while investigating the associations between normally distributed variables (TPV and NPV) and Spearman test was used for non-normally distributed variables (CPV). For all tests a significance level of 0.05 was used.

## Results

The population of this retrospective study was 167 patients. The mean age of all patients was 38.77 ± 23.2 years (median: 37 years; range between 0 and 80 years). 87 patients (52%) were male (mean age: 35.98 ± 23.06 years; median age: 32 years; range between 0 and 75 years), 80 patients (48%) were female (mean age: 41.8 ± 23.13 years; median age: 42 years; range between 4 and 80 years). The median values were 88.5 mm^3^ (12.3 mm^3^–411 mm^3^) for total pineal volume (TPV), 74.3 mm^3^ (12.3 mm^3^–298 mm^3^) for noncalcified pineal volume (NPV), and 3.9 mm^3^ (0 mm^3^–141 mm^3^) for calcified pineal volume (CPV). In both females and males, TPV, NPV, and CPV had high inter-individual variations in all age groups (p < 0.001 for TPV, CPV, POC and P = 0.03 for NPV, Table 1). POC showed a gradual increase from 0–49 years, but the values in higher age groups evolved inconsistently (Table 1). Also in the ≥70 group, when compared with the 60–69 age group, the calcified volume and POC value were significantly lower (P: 0.036, P: 0.034, respectively). When we analyzed the gender effect on pineal parenchyma between the ages of 10–39, although insignificant, the POC value of women was slightly higher then men. The mean CPV was higher in females in the 40–49 age group, but in contrast it was lower in the 60–69 age group when compared with men (P = 0.02 and P = 0.04, respectively) (Table 2).

**Table 1:** Distribution of the pineal volume parameters according to age groups. **N:** Number, **TPV:** Total pineal volume, **NPV:** Non-calcified pineal volume, **CPV:** Calcified pineal volume, **POC:** Proportion of calcification.

Age group	N	TPV	NPV	CPV	POC (%)

**0–9**	20	55.1 ± 18.69	55.04 ± 18.69	0.06 ± 0.22	0.1
**10–19**	22	86.15 ± 29.94	80.93 ± 28.14	5.22 ± 11.86	6.05
**20–29**	24	78.68 ± 19.3	69.56 ± 22.66	9.12 ± 17.6	11.59
**30–39**	20	93.24 ± 23.68	77.42 ± 23.04	15.81 ± 18.64	16.95
**40–49**	22	116.7 ± 44.51	83.68 ± 30.86	33.02 ± 42.41	28.29
**50–59**	18	104.69 ± 31.13	79.64 ± 31.87	25.05 ± 27.93	23.92
**60–69**	21	131.04 ± 88.55	94.15 ± 71.44	36.89 ± 33.47	28.15
**≥70**	20	101.73 ± 30.6	81.46 ± 32.11	20.27 ± 26.29	19.92
**Total**	**167**				
**p value**		**<0.001**	**0.03**	**<0.001**	**<0.001**

**Table 2:** Pineal volume parameters in relation to age and gender. The Pearson test was used for * total pineal gland volume (TPV), *** noncalcified pineal volume (NPV) and **** proportion of calcification (POC) between males and females. The Spearman test was used for ** calcified pineal volume (CPV).

	Male					Female					
Age group	N	TPV	CPV	NPV	POC (%)	N	TPV	CPV	NPV	POC (%)	p-value

0–9	10	58.18 ± 14.5	0.11 ± 0.31	58.07 ± 14.52	**0.18**	10	52.02 ± 22.50	0.1 ± 0.03	52.01 ± 22.50	**0.01**	0.48* 0.48** 0.68*** 0.73****
10–19	12	82.74 ± 28.74	1.97 ± 5.66	80.76 ± 29.67	**2.38**	10	90.25 ± 32.37	9.12 ± 16.07	81.13 ± 27.78	**10.1**	0.45* 0.72** 0.41*** 0.45****
20–29	14	76.52 ± 17.10	5.37 ± 12.24	71.15 ± 20.74	**7.01**	10	81.71 ± 22.63	14.36 ± 22.87	67.35 ± 26.11	**17.5**	0.43* 0.84** 0.5*** 0.47****
30–39	10	84.77 ± 19.79	12.83 ± 13.38	71.94 ± 14.49	**15.13**	10	101.71 ± 25.16	18.80 ± 23.12	82.91 ± 29.06	**22.46**	0.1* 0.48** 0.52*** 0.57****
40–49	12	122.76 ± 46.68	25.96 ± 39.85	96.80 ± 30.03	**21.14**	10	109.44 ± 43.04	41.49 ± 45.94	67.95 ± 24.81	**37.91**	0.62* 0.02** 0.49*** 0.34****
50–59	8	96.1 ± 30.18	24.38 ± 23.17	71.71 ± 27.99	**25.36**	10	111.57 ± 31.68	25.58 ± 32.49	85.99 ± 34.77	**22.92**	0.2* 0.36** 0.76*** 0.76****
60–69	11	96.56 ± 18.56	31.9 ± 20.79	64.66 ± 19.46	**33.03**	10	168.98 ± 118.28	42.39 ± 44.10	126.59 ± 93.22	**25.07**	0.13* 0.04** 0.97*** 0.42****
≥70	10	99.19 ± 31.17	22 ± 32.45	77.19 ± 34.37	**22.17**	10	104.28 ± 31.48	18.55 ± 20	85.73 ± 30.90	**17.78**	0.85* 0.73** 0.73*** 0.63****

## Discussion

Melatonin was firstly isolated by Lerner A. et al. in 1958 and in the literature it was described as the *fountain of eternal youth* [17,21]. Some studies found a correlation between pineal volume and age corresponding to melatonin secretion, which serves as a strong antioxidant against the effects of harmful compounds on the human body [9,10]. Intracranial calcifications are well known calcium and magnesium deposits that are called ‘brain sand’ in the pineal glands, mostly found in adult and aging patients [22]. According to the anatomical study by Allen et al., they found that the amount of calcified deposits increased in number and size in the aged pineal of senile rats [23].

Cross-sectional imaging studies have not yet provided elaborate evaluation of calcification of the pineal gland with thin section CT series to demonstrate calcification and noncalcified volume in healthy people [7,8]. A review of the literature provides little data about normal pineal volume and morphology. A possible explanation for the above mentioned contradiction in the age-related trends of pineal volume is the different methods used [9]. Studies in the literature provide contradicting data due to using a slice thickness of 4mm to 10mm in computed tomography. It is possible that they could not achieve optimal results in determining the rate of calcification of a tissue with an average size of 7.4mm in length, 6.9mm in width, and 2.5mm in height in autopsy series [24]. Scanning 22 pineal gland autopsy specimens in a skull phantom with different slice thicknesses, Schmitz SA. et al. found that slices as thin as 1mm must be used to measure the gland volume [25]. This is the first volumetric CT study that reflects the normal and calcified tissue proportion of pineal gland according to age groups, in which 0.5mm thick slices were used to achieve optimal measurements.

In the study by Tapp and Huxley, a gradual increase in the weight of the pineal gland from puberty to old age was demonstrated [26]. In a study by Sumida M. et al. in 1996, they analyzed the size of the normal pineal gland in 249 patients (129 male and 120 female) aged 2 weeks to 20-years-old. They calculated the volume of the pineal gland using a combination of sagittal, coronal, and axial magnetic resonance (MR) images with a slice thickness of 5mm. They found that the size of the pineal gland was significantly smaller in patients younger than 2-years-old compared to older patients. The size of the pineal gland increased until 2-years of age and remained stationary between the ages of 2 and 20 years.

There was a large variation in size among all age groups and no difference in size was noted between males and females [24]. Although not regular, in contrast with this data, we found an increase in the TPV and CPV from birth to adulthood. We added volumetric CT data on pineal volume and calcification and we also excluded the pineal recess on sagittal images, which was not mentioned in previous studies.

Computed tomography (CT) scans can better show calcification than MR imaging (MRI). Recently, radiological studies of the pineal gland have been mainly conducted by CT on the existence of pineal calcification and the proportion of calcification on axial CT images over different populations [3,4,5,6, 27]. In the study by Mahrlberg R et al., assessing the degree of calcification in the pineal gland of patients with Alzheimer’s disease, they used 8 mm axial CT slices [14]. We found a decrease in the ≥70 age group in the proportion of calcified tissue when compared to the 60–69 age group. In the postmortem study by Hasegawa et al., after the age of 90, they showed a significant decrease in calcified tissue. Some pineal glands of patients over 90-years-old showed no calcification and appeared indistinguishable from those of younger subjects. These results may demonstrate that human pineal glands do not necessarily calcify with involution [28]. We believe that investigations should focus on examining the volumetric analysis of active pineal tissue and the calcified portion according to serum and urine melatonin levels in patients of advanced age.

Our study has some limitations that may influence the results. Depending on the retrospective design of the study, we excluded some disorders according to patients’ hospital records, that may have a link with pineal failure. We couldn’t exclude phospho-calcic metabolism diseases according to these records in some patients. The enormous inter-individual variation of the pineal gland complicates radiological evaluations [7,8]. In addition, apart from calcification, pineal cysts may also act to reduce the active pineal volume that secretes melatonin. In morphometric analysis using high-resolution 3D sequences, true fast imaging with steady state precession (true FISP) MRI sequence with 0.9mm slices, by Bumb JM et al., volumetry of 347 patient pineal glands were analyzed. Of the 347 pineal glands, 40.3% contained cysts. The study group did not consist of only healthy people and the patients had various intracerebral diseases, such as intracranial neoplasms, ischemia, hemorrhage, inflammatory disease of the central nervous system, epilepsy, and hydrocephalus, and just 16.1% had normal findings, so this may potentially affect the results of the study [29].

In the study by Nolte et al., they evaluated 15 healthy male subjects’ (20–27 years) pineal parenchyma volume and circadian melatonin profiles, 24-hour melatonin per volume of pineal tissue and melatonin profiles of cystic and solid glands. They found that parenchyma volume correlates linearly to melatonin plasma concentration. So pineal cysts may have a negative effect on melatonin secretion by compressing the parenchyma and, therefore, the pinealocytes [30]. According to another study by the same author, analyzing pineal cysts of 111 patients with MRI, they showed that 35.1% of the study group had cysts [31]. We may have excluded some of the pineal cysts from the pineal parenchyma, especially the large ones from the cerebrospinal fluid in the neighboring third ventricle because of their low density.

A previous study by Liebrich LS et al., compared melatonin levels in saliva and pineal volume by means of three-dimensional T2 turbo spin echo (3D-T2-TSE)—solid parenchyma—and susceptibility-weighted imaging (SWI, exclusion of calcified areas) in a population of 103 young and healthy participants. Results of this study demonstrated a negative correlation between pineal volume and sleep rhythm disturbances [32]. Using higher resolution (0.6 mm, isotropic) and higher tissue contrast in 3D-T2-TSE with the addition of SWI seems to provide an ideal measurment of solid, melatonin secreting pineal parenchyma. This method can be used in larger populations with the aim of introducing the relation between age and solid pineal parenchyma volume. Although it is commonly known that SWI is a very sensitive sequence to detect calcification, there is no information regarding the correlation between the amount of calcification and the signal intensity in SWI. Further studies are needed to clarify this question.

We assume that optimized volumetry of active pineal tissue and therefore a higher correlation of melatonin and pineal parenchyma can potentially be improved by a combination of MR and CT imaging in addition to serum melatonin levels. Moreover, in order to improve MR quantification of pineal calcifications, the combined approach would possibly allow an optimization and calibration of MRI sequences by CT and then perhaps even make CT unnecessary [31]. In addition, our study group was not large enough to reflect data of the whole community.

## Conclusion

Calcifications of the pineal gland and decreased production of melatonin have the same trend. This study brings provides a comprehensive assessement of the pineal gland morphology on imaging in different age groups. Future studies are necessary to verify our results in a large population of advanced age to reach stronger statistical association between the pineal gland morphology and function.

## Competing Interests

The authors declare that they have no competing interests.
